# Characteristics of interval gastric neoplasms detected within two years after negative screening endoscopy among Koreans

**DOI:** 10.1186/s12885-021-07929-y

**Published:** 2021-03-02

**Authors:** Joo Hyun Lim, Ji Hyun Song, Su Jin Chung, Goh Eun Chung, Joo Sung Kim

**Affiliations:** 1 Department of Internal Medicine, Healthcare Research Institute, Healthcare System Gangnam Center, Seoul National University Hospital, 152, Teheran-ro, Gangnam-gu, Seoul, 06236 South Korea; 2 Department of Internal Medicine and Liver Research Institute, Seoul National University College of Medicine, 101, Daehak-ro, Jongno-gu, Seoul, 03080 South Korea

**Keywords:** Gastric neoplasm, Screening endoscopy, *H. pylori*, Atrophic gastritis, Undifferentiated histology

## Abstract

**Background:**

In Korea, where gastric cancer is highly prevalent, biennial endoscopy is recommended among individuals over 40. Even under regular screening, some are still diagnosed at advanced stages. We aimed to identify characteristics of interval gastric neoplasms (IGNs) with rapid progression.

**Results:**

Newly-diagnosed gastric neoplasms detected in screening endoscopy between January 2004 and May 2016 were reviewed. Among them, those who had previous endoscopy within 2 years were enrolled. Endoscopic findings, family history of gastric cancer, smoking, and *H. pylori* status were analysed. Totally, 297 IGN cases were enrolled. Among them, 246 were endoscopically treatable IGN (ET-IGN) and 51 were endoscopically untreatable IGNs (EUT-IGN) by the expanded criteria for endoscopic submucosal dissection. Among EUT-IGNs, 78% were undifferentiated cancers (40/51) and 33% showed submucosal invasion (13/40). They were median 2.0 cm in size and more commonly located in the proximal stomach than ET-IGNs (70.6% vs. 41.9%, *p* < 0.001). EUT-IGN was independently related with age < 60 (OR, 2.09; 95%CI, 1.03–4.26, *p* = 0.042), *H. pylori* (OR, 2.81; 95%CI, 1.20–6.63, *p* = 0.018), and absent/mild gastric atrophy (OR, 2.67; 95%CI, 1.25–5.72, *p* = 0.011). Overall and disease-specific survival were not significantly different between the two groups, however EUT-IGN tended to have short disease-specific survival (overall survival, *p* = 0.143; disease-specific survival, *p* = 0.083).

**Conclusions:**

Uniform screening endoscopy with two-year interval seems not enough for rapid-growing gastric neoplasms, such as undifferentiated cancers. They tended to develop in adults younger than 60 with *H. pylori* infection without severe gastric atrophy. More meticulous screening, especially for proximal lesions is warranted for adults younger than 60 with *H. pylori* infection before development of gastric atrophy.

**Supplementary Information:**

The online version contains supplementary material available at 10.1186/s12885-021-07929-y.

## Background

Although its incidence and mortality are continuously decreasing, gastric cancer is still the 5th most common cancer and the 3rd leading cause of cancer-related death worldwide. Annually, more than a million new gastric cancer cases arise and more than 780,000 gastric cancer-related deaths occur over the world [1]. Especially in East Asia, gastric cancer is one of the greatest concerns due to the extremely high incidence [2–4]. In Korea, although the incidence of *Helicobacter pylori* (*H. pylori*) infection, which is one of the greatest risk factors for gastric cancer, has been gradually decreased, showing 66.9% in 1998, 59.6% in 2005, 54.4% in 2011, and 43.9% in 2016 [5], gastric cancer is still the most common cancer and its age-standardised rate reached up to 33.3 in 2017 statistics [6]. The National Cancer Screening Program in Korea, which covers biennial upper gastrointestinal endoscopy for those over 40 years old, has been in place since 2004 [7, 8]. Since then, the five-year survival rate of gastric cancer in Korea has considerably increased, which although is still an unsatisfactory rate at less than 80% [9]. This can be attributed in part to insufficient endoscopic screening rate of only about 60% [10]. However, even despite regular screening, some are still diagnosed with advanced diseases.

Interval cancer is defined as cancer detected between regular screenings, which can be a great setback to the cancer screening. The concept of “interval cancer” is well established in colorectal cancer, as a quality indicator of screening [11]. However, little has been investigated about interval gastric cancer. This terminology of “interval gastric cancer” is occasionally used only in Far East where nationwide endoscopic screening for gastric cancer is established [12]. Interval cancer can be missed lesion or true new lesion. Qualified endoscopy may reduce missing rate, however, new lesions with rapid progression are inevitable. Also, whether the two-year interval of gastric cancer screening is optimal is still on debate. Several studies have reported better outcome of endoscopic screening with less than 2 years of interval [13–15]. In current study, we defined “interval gastric neoplasm (IGN)” as gastric neoplasm including dysplasia and cancer detected within 2 years after negative screening. We assumed that better understanding on IGN will enable better tailored-screening for gastric cancer. This study aimed to identify characteristics of IGN detected during regular screening and risk factors for rapid progression.

## Methods

### Patients

We retrospectively reviewed medical records of patients who were newly diagnosed with gastric neoplasms, including dysplasia and cancer, during screening endoscopy at a single health-checkup center between January 2004 and May 2016. Among them, those who underwent previous endoscopy within 2 years were enrolled. Endoscopy was performed with Fujinon VP-XL 402/4400 until 2009 and Olympus CV-260SL/290 thereafter. In order to identify potential risk factors for rapid progression, we reviewed the following data: endoscopic findings, family history of gastric cancer within 1st degree relatives, smoking history (never vs. former/current), and *H. pylori* infection status. Those with either anti-*H. pylori* IgG antibody sero-positivity, positive result in rapid urease test, or histologic findings of *H. pylori* in Giemsa staining were considered to have *H. pylori* infection. Anti-*H. pylori* antibody was tested with enzyme-linked immunosorbent assay (*H. pylori*-EIA-Well, Radim, Rome, Italy) until March 2013 and with chemiluminescent microparticle immunoassay (Immulite 2000 CMIA, Siemens, UK) since April 2013. Anti-*H. pylori* IgG levels higher than 30 U/mL by EIA and 1.10 IU/mL by CIMA were regarded positive. We also reviewed the previous endoscopic images to check if any abnormality had been found at the same location where the neoplasm developed. The Institutional Review Board of our center, which complies with the declaration of Helsinki, approved this study (IRB No. H-1504-045-663). Patient consent was waived because of the retrospective nature of this study.

### Definitions

In this study, endoscopically treatable-IGN (ET-IGN) was defined as adenoma or gastric cancer detected within 2 years after negative screening endoscopy, which is within the expanded criteria for endoscopic submucosal dissection (ESD), except for undifferentiated histology [16]. This includes low- to high-grade dysplasia regardless of size of the lesion or well- to moderately-differentiated adenocarcinoma. As for adenocarcinoma, following three size criteria were included: mucosal cancer without ulceration regardless of size, mucosal cancer < 3 cm with ulceration, or submucosal cancer < 3 cm with SM1 invasion.

Endoscopically untreatable-IGN (EUT-IGN) was defined as gastric cancer detected within 2 years after negative screening endoscopy, which is over the expanded criteria for ESD, including undifferentiated histology regardless of size.

Severity of gastric atrophy was defined using the Kimura-Takemoto classification designed for the categorisation of endoscopic atrophic gastritis [17] by three expert endoscopists (JHL, JHS, SJC) under mutual discussion. Intestinal metaplasia was defined as gross endoscopic finding with ash-colored mucosal nodularity.

Biopsy specimens were examined and reported according to the WHO criteria [18]. As for gastric cancer, well-to-moderately differentiated tubular adenocarcinoma or papillary adenocarcinoma was classified as differentiated type, while poorly differentiated adenocarcinoma, signet-ring cell carcinoma (poorly cohesive carcinoma), or mucinous carcinoma was classified as undifferentiated type.

### Statistical analysis

For categorical variables, Chi-square test or Fisher’s exact test was applied, while Student *t*-test was used to compare continuous variables. Multivariable analysis was performed by using logistic regression analysis with potential risk factors found in univariable analyses with clinical relevance. Survival analysis was performed with log-rank test and Cox proportional hazard model. All the analyses were performed with the Statistical Package for the Social Sciences, version 23.0 for Windows (SPSS, Seoul, Korea). All tests were two-sided and *p* values < 0.05 were considered statistically significant.

## Results

### Baseline clinical characteristics

**Fig. 1:**
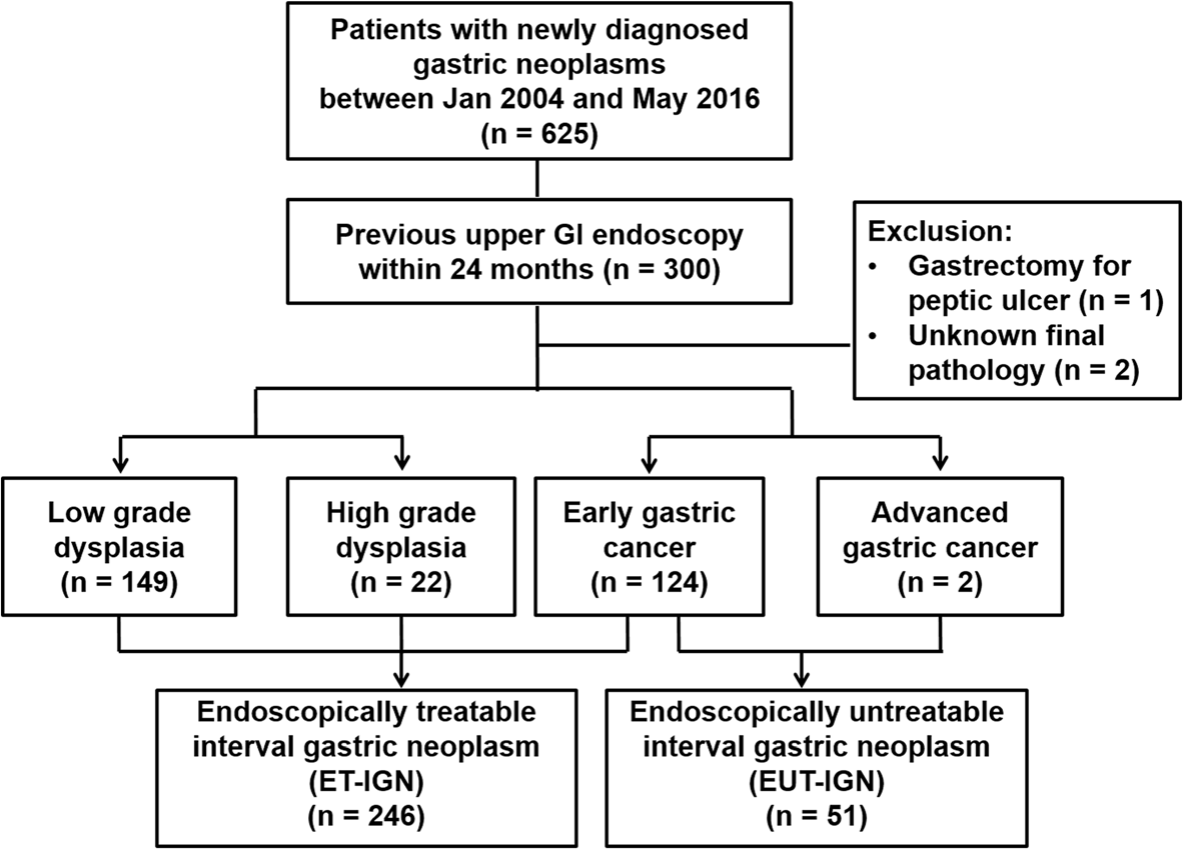
Study flow chart showing patient enrollment

Overall, 625 patients who were newly diagnosed with gastric neoplasms were reviewed (Fig. 1). Among them, 300 patients who underwent previous upper gastrointestinal endoscopy within 24 months were included. One patient who had previous gastrectomy for peptic ulcer and two patients with unclear final pathology were excluded, and the remaining 297 individuals were finally enrolled. Among the total 297 lesions in 297 individuals, 149 were diagnosed with low-grade dysplasia, 22 with high-grade dysplasia, 124 with early gastric cancer (EGC), and 2 with advanced gastric cancer (AGC). Altogether, 246 cases were ET-IGNs and 51 cases were EUT-IGNs. There has been no one with synchronous lesions in this study.

Overall mean age was 59.4 and the EUT-IGN group was younger than the ET-IGN group (55.0 ± 8.5 vs 60.3 ± 9.0, *p* < 0.001, Table 1). Between the two groups, the EUT-IGN group had less proportion of males (58.8% vs 79.7%, *p* = 0.001) and smaller body mass index (BMI) at diagnosis than the ET-IGN group. Past history of cancer other than gastric cancer was not significantly different between the two groups. Overall family history of gastric cancer was found in 22.6% and it was not different between the two groups. Overall *H. pylori* infection rate was 71.6% and which was higher in EUT-IGN group, although without statistical significance (82.4% vs. 69.4%, *p* = 0.062). Smoking rate was significantly higher in the ET-IGN group (69.5% vs 49.0%, *p* = 0.005). The *H. pylori* infection rate among IGNs were higher than that among the general Korean population (59.6% in 2005; 54.4% in 2011; 43.9% in 2016) [5]. However, despite the decreasing general trend of *H. pylori* infection rate, the rate among IGN rather somewhat increased although without statistical significance (69.6% in 2004–2008; 68.2% in 2009–2013; 78.3% in 2014–2016; *p* = 0.231). Among the total 297 patients, about 10% (*n* = 31) had abnormal lesion at the same location in the previous endoscopy, however, the biopsies all revealed negative result for neoplasm, only with the finding of active gastritis. However, the rate of previous abnormal lesion at the same location was not different between the two groups (13.7 and 9.8% in the EUT-IGN and the ET-IGN groups, respectively, *p* = 0.399). Atrophic gastritis and intestinal metaplasia were significantly more common in the ET-IGN group than in the EUT-IGN group. Also, moderate to severe atrophic gastritis was more frequent in the ET-IGN group (81.7% vs 49.1%, *p* = 0.002).

**Table 1:** Demographic and clinical characteristics of the study patients

	Overall (***n*** = 297)	ET-IGN group (***n*** = 246)	EUT-IGN group (***n*** = 51)	***p***^†^
**Age, mean ± SD (yr)**	59.4 ± 9.1	60.3 ± 9.0	55.0 ± 8.5	< 0.001
**Male sex (%)**	226 (76.1)	196 (79.7)	30 (58.8)	0.001
**BMI, median (IQR) (kg/m**^**2**^**)**	24.4 (22.4–26.0)	24.6 (22.6–26.0)	23.5 (21.3–25.8)	0.030
**Previous diagnosis with other cancer (%)**	37 (12.5)	31 (12.5)	6 (11.8)	0.869
**Family history of gastric cancer (%)**	67 (22.6)	53 (21.5)	14 (27.5)	0.358
***H. pylori*** **infection**^‡^ **(%)**	212/296 (71.6)	170/245 (69.4)	42/51 (82.4)	0.062
**Current or ex-smoker (%)**	196 (66.0)	171 (69.5)	25 (49.0)	0.005
**Previous abnormality at the same location (%)**	31 (10.4)	24 (9.8)	7 (13.7)	0.399
**Gastric atrophy, Kimura-Takemoto classification (%)**				0.002
**Absent (C0)**	12 (4.0)	7 (2.8)	5 (9.8)	
**Mild (C1, C2)**	54 (18.2)	38 (15.4)	16 (31.4)	
**Moderate (C3, O1)**	165 (55.6)	141 (57.3)	24 (47.1)	
**Severe (O2, O3)**	66 (22.2)	60 (24.4)	6 (11.8)	
**Intestinal metaplasia**^§^ **(%)**	175 (58.9)	156 (63.4)	19 (37.3)	0.001
**Interval since last upper GI endoscopy, median (IQR) (months)**	12.0 (11.0–15.0)	12.0 (11.0–15.0)	12.0 (11.0–14.0)	0.847

*ET-IGN* endoscopically treatable gastric neoplasm; *EUT-IGN* endoscopically untreatable gastric neoplasm; *SD* standard deviation; *BMI* body mass index; *IQR* interquartile range; *H. pylori*, *Helicobacter pylori*; *GI* gastrointestinal

^†^ Comparison between ET-IGN and EUT-IGN

^‡^ Some denominators do not match the total numbers because of missing data

^§^ Endoscopically diagnosed intestinal metaplasia

Continuous variables are presented as the mean ± standard deviation or median with interquartile range according to fitness of normal distribution and categorical variables are presented as numbers and effective percentage excluding missing data

### Pathological characteristics of interval neoplasms

Table 2 shows the pathological characteristics of 297 IGNs. Overall median size was less than 1.0 cm, while the median size of EUT-IGNs was as large as 2.0 cm. Ulceration was ten times more commonly found in the EUT-IGN group than in the ET-IGN group (31.4% vs 3.3%, *p* < 0.001). Undifferentiated histology accounted for 80% of the EUT-IGNs, and submucosal invasion was detected in about one-third of the EUT-IGNs (13/40, 32.5%). ET-IGNs were most commonly found in the lower third of the stomach and then in the middle and upper third, whereas EUT-IGNs were commonly found in the order of the upper, middle, and lower third.

**Table 2:** Pathologic characteristics of interval neoplasms

	Overall (***n*** = 297)	ET-IGN (***n*** = 246)	EUT-IGN (***n*** = 51)	***p***^†^
**Size, median (IQR) (cm)**	0.90 (0.60–1.60)	0.80 (0.50–1.20)	2.00 (1.40–3.00)	< 0.001
**Ulcer (%)**	24 (8.1)	8 (3.3)	16 (31.4)	< 0.001
**Undifferentiated histology (%)**	40 (13.5)	0 (0.0)	40 (78.4)	–
**Submucosal invasion**^‡^ **(%)**	14/282 (5.0)	1/242 (0.4)	13/40 (32.5)	< 0.001
**Location (%)**				< 0.001
**Upper third**	58 (19.5)	39 (15.9)	19 (37.3)	
**Middle third**	81 (27.3)	64 (26.0)	17 (33.3)	
**Lower third**	158 (53.2)	143 (58.1)	15 (29.4)	

*ET-IGN* endoscopically treatable gastric neoplasm; *EUT-IGN*, endoscopically untreatable gastric neoplasm; *IQR* interquartile range

^†^ Comparison between ET-IGN and EUT-IGN

^‡^ Some denominators do not match the total numbers because of missing data

Continuous variables are presented as the median with interquartile and categorical variables are presented as numbers and effective percentage excluding missing data

In this study, there were two cases of AGC (Table 3). Both were female nonsmokers with undifferentiated histology and both lesions were located in the upper third of the stomach. One had a 15.5 cm-sized lesion, with linitis plastica. In retrospective review, similar fold thickening was found at the same location of the previous endoscopic images, which was found to be overlooked at that time after a negative result using forceps-biopsy. The other case was detected with only 1.5 cm-sized ulcero-fungating lesion with non-specific findings from the previous endoscopy. Diffuse peritoneal seeding was found at the time of staging workup and this patient died after 14.8 months later.

**Table 3:** Profiles of patients with AGC detected in less than two-year of interval

Age	Sex	FHx of gastric cancer	***H. pylori*** infection	Smoking	Atrophy	Intestinal metaplasia	Size	Ulcer	Histology	Location	Distant metastasis	Primary treatment modality	Recurrence	Survival duration (months)
35	F	None	Unknown	Never	Mild	No	Grossly 1.5 cm	Yes	Adenocarcinoma P/D with SRC component	Upper third	Peritoneum	Chemotherapy	–	14.8
59	F	None	Yes	Never	Moderate	Yes	15.5 cm	None	Adenocarcinoma P/D	Upper third	None	Total gastrectomy	Yes	23.4

*AGC* advanced gastric cancer; *FHx* family history; *H. pylori*, *Helicobacter pylori*; *P/D* poorly differentiated; *SRC* signet ring cell

### Multivariable analysis for risk of EUT-IGN

**Fig. 2:**
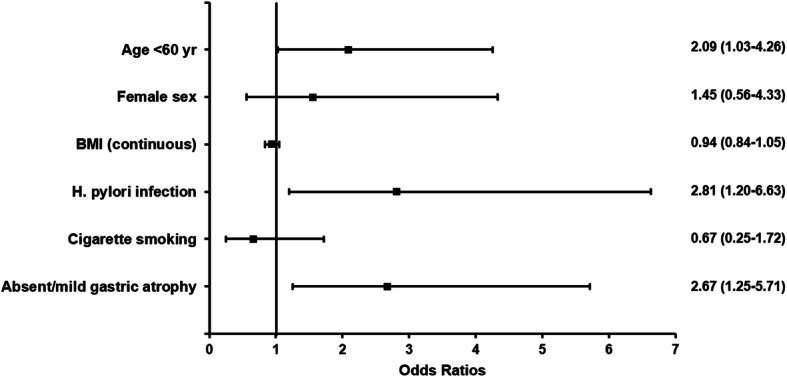
Odds ratios and 95% confidence intervals of potential risk factors for endoscopically untreatable-gastric neoplasm. BMI, body mass index; *H. pylori*, *Helicobacter pylori*

We performed multivariable analysis to evaluate the risk for EUT-IGN with potentially associated variables which showed *p* values < 0.10 in the univariable analyses (Fig. 2). In this analysis, age < 60 [odds ratio (OR), 2.09; 95% CI, 1.03–4.26; *p* = 0.042], *H. pylori* infection (OR, 2.81; 95% CI, 1.20–6.63; *p* = 0.018), and absent/mild atrophy (OR, 2.67; 95% CI, 1.25–5.71; *p* = 0.011) showed independent association with EUT-IGN under adjustment. Meanwhile, female sex (OR, 1.56; 95% CI, 0.56–4.33; *p* = 0.397), BMI (OR, 0.94; 95% CI, 0.84–1.05; *p* = 0.272), and smoking (OR, 0.66; 95% CI, 0.25–1.72; *p* = 0.398) were not independently related with EUT-IGN.

### Survival analysis of the IGNs

**Fig. 3:**
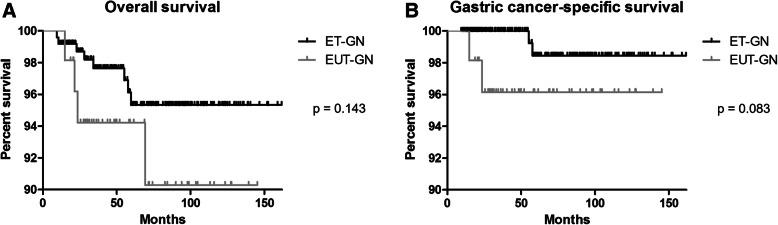
Kaplan-Meier curves comparing ET-IGN and EUT-IGN: (**a**) Overall survival; (**b**) Gastric cancer-specific survival. ET-IGN, endoscopically treatable-gastric neoplasm; EUT-IGN, endoscopically untreatable-gastric neoplasm

During the median follow-up duration of 58.4 months (interquartile range, 30.3–94.5), eight cases of death occurred in the ET-IGN group and four in the EUT-IGN group. Among them, two in each group were gastric cancer-related deaths. The detailed profiles of the two gastric cancer-related deaths developed among ET-IGN group are described in Table 4. Overall survival showed a tendency of lower survival in the EUT-IGN group than ET-IGN group, however, this failed to show statistical significance (*p* = 0.143, Fig. 3a). Under Cox proportional hazard model, EUT-IGN showed HR of 2.34 with 95% CI between 0.72 and 7.94, compared with ET-IGN. Meanwhile, gastric cancer-specific survival also showed a tendency of lower rate in EUT-IGN than in ET-IGN, with relatively near significance (*p* = 0.083, Fig. 3b). Under Cox proportional hazard model, EUT-IGN showed HR of 4.804 with 95% CI between 0.68 and 34.14.

**Table 4:** Profiles of the two cases of gastric cancer-related death developed after ESD for ET-GNs

Age at diagnosis	Sex	FHx of gastric cancer	***H. pylori*** infection	Smoking	Atrophy	Intestinal metaplasia	Size	Ulcer	Histology and stage	Location	Resection margin involvement	Months until recurrence	Recurrence involvement organ	Histology of recurrence
66	M	Yes	Yes	Ex-smoker	Severe	Yes	4.2 cm	None	Tubular adenoma, high grade	Middle third	No	45	Stomach, liver	ADC M/D
61	M	None	Yes	Ex-smoker	Moderate	Yes	1.4 cm	Yes	ADC M/D, T1b	Lower third	No	47	Liver	ADC

*FHx* family history; *H. pyloriHelicobacter pylori*; *ADC* adenocarcinoma; *M/D* moderately differentiate

### Subgroup analysis among interval gastric cancers (IGCs)

We also performed subgroup analysis among IGCs excluding gastric dysplasia. Among them, endoscopically untreatable–IGCs (EUT-IGCs) were younger with larger composition of females, and less likely smokers (Supplementary Table 1). Also EUT-IGCs were less likely to have moderate-to-severe atrophy or intestinal metaplasia compared with endoscopically treatable-IGCs (ET-IGCs). Obviously, EUT-IGCs were more likely to have ulcers and larger lesions with submucosal invasion.

In multivariable analysis, age (OR, 0.94; 95% CI, 0.89–0.99; *p* = 0.01), female gender (OR, 4.00; 95% CI, 1.43–11.14; *p* = 0.01), and absent/mild atrophy (OR, 2.96; 95% CI, 1.08–8.07; *p* = 0.03) were shown to be independently associated with EUT-IGC. Although without statistical significance, those with abnormalities found at the same location in the previous exam were shown to have high tendency to develop EUT-IGC (OR, 4.29; 95% CI, 0.86–21.26; *p* = 0.08).

In terms of survival, EUT-IGCs were more likely to have poorer overall survival and gastric cancer-specific survival, although without statistical significance (Supplementary Fig. 1A and 1B).

### Subgroup analysis among IGNs detected since 2010

As the scope used in the screening has been improved over time, the endoscopic images taken before 2010 were of poor quality with low resolution compared with those taken thereafter with new Olympus scope. Therefore, we performed subgroup analysis among IGNs detected since 2010. Since 2010, 204 IGNs were detected including 170 ET-IGN and 34 EUT-IGN. Their baseline and pathologic characteristics were not much different from those of overall subjects (Supplementary Table 2). In multivariable analysis, age < 60 (OR, 0.94; 95% CI, 0.90–0.99; *p* = 0.02), *H. pylori* infection (OR, 3.92; 95% CI, 1.23–12.46; *p* = 0.02), and absent/mild atrophy (OR, 3.30; 95% CI, 1.25–8.72; *p* = 0.02) were revealed as independent risk factors for EUT-IGN, just like in the overall subjects.

## Discussion

Overall, among the 297 patients with IGNs diagnosed by screening endoscopy, 17% had EUT-IGN and most of them were undifferentiated adenocarcinoma. After multivariable analysis, age < 60, *H. pylori* infection, and absent/mild atrophic gastritis were shown to be associated with EUT-IGN among health screening population. Also, patients with EUT-IGN showed a tendency of lower gastric cancer-specific survival compared with those with ET-IGN.

In this study, IGN was defined as gastric dysplasia or cancer diagnosed between biennial endoscopic screenings. As the concept of interval cancer is not widely used for gastric cancer, there have been little references regarding this. Several previous studies dealt with interval gastric cancer [19, 20], however our study is somewhat different from those studies. Usually, the concept interval cancer is used for quality indicator of screening. This means that it is highly probable that interval cancer is a missed lesion in the previous screening. However, interval cancer might be a newly developed lesion after a negative screening, as well. Unlike previous studies, we focused on the latter possibility, as we all reviewed previous endoscopic images and confirmed no abnormal findings. Also, considering the highly improved 5-year survival rate of gastric cancer, we need to more focus on the quality of life rather than mortality now-a-days. Whether to treat with surgery or endoscopic resection is one of the great component deciding the quality of life. Therefore, we also included dysplasia to further investigate the adequacy of 2 year interval and risk for rapid progression which disables endoscopic resection. In this concept the new definition of “IGN” was made.

It is well known that gastric cancer prognosis highly depends on the stage at diagnosis [21]. Also, it is widely accepted that EGC which is defined as gastric cancer confined to mucosa has very low rate of lymph node invasion [22] and is related with excellent 5-year survival rate compared with AGC [23]. Therefore gastric cancer is often divided into EGC and AGC considering its different treatment modality and prognosis. Also, gastric dysplasia has been widely accepted as precancerous lesion, since Correa et al. hypothesised the gastric carcinogenesis cascade which includes the serial progression of chronic inflammation into atrophic gastritis, intestinal metaplasia, gastric dysplasia, and finally gastric adenocarcinoma [24]. Therefore, we further divided IGN into ET-IGN and EUT-IGN according to the expanded criteria for ESD. Since ESD was first introduced in the 1990s, it has largely replaced the role of radical gastrectomy for EGC without lymph node metastasis. These days, ESD is established as one of the standard treatment modalities for early gastric neoplasm because it enables en bloc resection with less invasiveness than conventional surgery [25–27]. The quality of life in gastric cancer patients largely depends on whether endoscopic treatment is possible or not. With the prevalence of endoscopic screening, the proportion of early-stage diagnosis of gastric neoplasm increases, and thus the role of endoscopic treatment such as ESD is continuously growing as well. With the development of diagnostic and therapeutic technology, the EGC diagnosis rate nowadays reaches up to 80% in countries with high incidence rates of gastric cancer like Korea and Japan [14, 28]. Thus we further sub-classified gastric neoplasms according to the criteria for ESD to better reflect the prognosis and quality of life in patients with IGNs.

In this study, the clinicopathological characteristics of IGNs were not different from those of general gastric cancers in terms of mean age, male predominance, and the rate of *H. pylori* infection [1]. Meanwhile, patients with EUT-IGNs showed a significant difference; they were younger, with relatively higher proportion of female, with lower smoking rate. They had lower grade of atrophic gastritis and less likely to have intestinal metaplasia, despite higher rate of *H. pylori* infection and which are similar with the characteristics seen in diffuse type gastric cancer. Diffuse type gastric cancer is also related with younger age, rapid progression [29], and higher rate of *H. pylori* infection [30]. On the other hand, intestinal type GC is known to be more associated with multi-step carcinogenic cascade of chronic inflammation which comprises atrophic gastritis, intestinal metaplasia, and dysplasia [24, 31, 32]. In our study, among the EUT-IGNs, 80% were undifferentiated gastric cancers with Lauren diffuse type. As for the tumor location, EUT-IGNs showed tendency to be located at the upper or middle third of the stomach, while ET-IGNs were most commonly found at the lower third. This was also in line with the results of previous research which compared diffuse and intestinal type EGCs [30]. The relationship between *H. pylori* infection and the upper third gastric cancer has been controversial. However, a previous meta-analysis on this issue has shown that the relationship between cardia cancer and *H. pylori* infection is highly positive in Eastern countries where gastric cancer incidence is high, while it tends to be neutral or even negative in Western countries with low gastric cancer prevalence [33]. This phenomenon might be attributed to the co-existence of two distinct types of cancers, one similar to *H. pylori-*related gastric cancer and the other similar to acid/bile-related esophageal cancer, which are prevalent in Eastern and Western countries, respectively. Since the upper part of the stomach is usually observed less thoroughly than the antrum, there might have been missed lesions during the previous endoscopic examination. However, on the review of the previous endoscopic images, any abnormalities at the same location were all confirmed to be free of neoplastic change. Only one case with diffuse fold thickening in the previous exam, which showed negative result from the forcep biopsy was finally diagnosed as AGC with linitis plastica by using CT scan after the next screening endoscopy. This case gives a lesson that conscious evaluation is needed for cases suspicious for Bormann type IV AGCs. The two cases of interval AGCs in this study both survived less than 2 years since the diagnosis. The other one than the case of Bormann type IV gastric cancer was diagnosed with relatively small lesion, however revealed peritoneal seeding and was unable to undergo curative treatment. These cases show that regular biennial endoscopy cannot be the perfect screening for gastric cancer in a high prevalence area and customised screening or effective way of prevention is needed for high risk individuals.

In multivariable analysis, age < 60, *H. pylori* infection and absent/mild atrophic gastritis were demonstrated to be independent risk factors for EUT-IGN. Also, young age, female gender, and absent/mild atrophic gastritis were shown to be related with EUT-IGC in subgroup analysis among IGCs. Such factors are all known risk factors for the diffuse type gastric cancers [29, 30]. This means that endoscopic screening with two-year interval may not be enough for rapidly growing types of gastric cancers including those with undifferentiated histology. In current study, we did not count undifferentiated gastric cancers as the expanded criteria for ESD. Some researchers consider undifferentiated gastric cancers < 2 cm as a possible candidate for ESD [22, 23], however there are still controversy on this issue due to the possibility of lymph node metastasis [34, 35]. In this study, undifferentiated cancers were all included in the EUT-IGN group regardless of size. Two-thirds of undifferentiated cancers were larger than 2 cm, showing the rapid progression of undifferentiated cancers. Recent studies have reported that the long-term outcome of ESD was not different from that of surgery among undifferentiated gastric cancers < 2 cm [36, 37]. Taking this into account, under the assumption that undifferentiated cancers < 2 cm can be established as candidate for ESD, more intensive screening endoscopy less than two-year interval would be helpful for a high-risk group of undifferentiated gastric cancer. There have been several studies suggesting endoscopic screening with less than 2 years of interval [13–15]. We suppose our current result is in line with these studies in that it showed insufficiency of uniform biennial screening for early detection of gastric neoplasms.

Another solution for prevention of IGN, which is more likely to be acceptable in clinical practice would be extensive *H. pylori* eradication in adults, especially those younger than 60 years old. In our results, EUT-GN showed a tendency of lower disease-specific survival and included AGC cases. In general, gastric cancers in young ages are more likely to be aggressive and diagnosed at advanced stages. Therefore, *H. pylori* eradication for young adults regardless of the existence or severity of gastric atrophy might be helpful to reduce mortality from gastric cancer in a certain sub-population.

In this study, the median interval since last endoscopy was 12 months. This means that most of the enrolled subjects underwent endoscopy annually, showing that interval cancer can occur despite annual endoscopy. This is the reason why we designed this study. Some people develop gastric cancer very rapidly and even some skip the premalignant steps such as atrophy/intestinal metaplasia or dysplasia. Therefore, we performed subgroup analysis among only cancers excluding dysplasia, and where we found that IGC shows similar characteristics with IGN.

There are several limitations in this study. First, we did not compare the characteristics of EUT-IGN with healthy control, which made it unable to evaluate the general risk factors for EUT-IGN. However, healthy control is not considered indispensable since the risk factors for overall gastric cancer is already well-known. Second, we did not investigate gastrointestinal symptoms of the participants. The reason why these participants underwent screening within 2 years was not achieved. However, even though the recommended gastric cancer screening interval is 2 years by the National Cancer Screening Program in Korea, opportunistic screening is frequently performed with a shorter interval due to either the screenee’s preference or previously defined risk factors. Third, as our study included subjects over more than a decade of time, there were technical improvement in the endoscopic device. However, we could not adjust this factor.

## Conclusions

Considering that one out of six IGNs were EUT-IGNs, current uniform screening with biennial endoscopy seems not enough to improve the prognosis of rapid growing neoplasms such as undifferentiated cancers. Also such rapid growing neoplasms tended to show relatively low disease specific survival. Therefore more meticulous and high-quality screening endoscopy which include close observation especially at proximal area with enough time should be applied for adults, especially those younger than 60 years old with *H. pylori* infection before the development of severe gastric atrophy.

## Supplementary Information

**Additional file 1 Fig. S1** Kaplan-Meier curves comparing ET-IGC and EUT-IGC: (a) Overall survival; (b) Gastric cancer-specific survival. ET-IGC, endoscopically treatable-gastric cancer; EUT-IGC, endoscopically untreatable-gastric cancer.

**Additional file 2 Table S1**. Baseline and pathologic characteristics of interval gastric cancer. **Table S2**. Baseline and pathologic characteristics of interval gastric neoplasms detected since 2010.

## Data Availability

The datasets used and/or analysed during the current study are available from the corresponding author on reasonable request.

## Abbreviations

IGN: Interval gastric neoplasm
*H. pylori*: *Helicobacter pylori*
ET-IGN: Endoscopically treatable-IGN
ESD: Endoscopic submucosal dissection
EUT-IGN: Endoscopically untreatable-IGN
EGC: Early gastric cancer
AGC: Advanced gastric cancer
BMI: Body mass index
OR: Odds ratio
IGC: Interval gastric cancer
EUT-IGC: Endoscopically untreatable-IGC
ET-IGC: Endoscopically treatable-IGC
