# Supplementary material for: Characteristics of interval gastric neoplasms detected within two years after negative screening endoscopy among Koreans
Source: BMC Cancer. 2021 Mar 2;21:218. doi: 10.1186/s12885-021-07929-y (PMC7923316; doi:10.1186/s12885-021-07929-y)
Supplement: Supplementary file 1 — Additional file 1 Fig. S1 Kaplan-Meier curves comparing ET-IGC and EUT-IGC: (a) Overall survival; (b) Gastric cancer-specific survival. ET-IGC, endoscopically treatable-gastric cancer; EUT-IGC, endoscopically untreatable-gastric cancer. [file 12885_2021_7929_MOESM1_ESM.docx]

**Supplementary table 1. Baseline and pathologic characteristics of interval gastric cancer**

|  | **Overall**  **(n = 126)** | **ET-IGC group**  **(n = 75)** | **EUT-IGC group**  **(n = 51)** | ***p***^†^ |
| --- | --- | --- | --- | --- |
| **Age, mean ± SD (yr)** | 58.5 ± 9.3 | 60.9 ± 9.1 | 55.0 ± 8.5 | <0.001 |
| **Male sex (%)** | 96 (76.2) | 66 (88.7) | 30 (58.8) | 0.001 |
| **BMI, median (IQR) (kg/m^2^)** | 24.1 (22.3-26.4) | 24.7 (22.7-26.6) | 23.5 (21.3-25.8) | 0.151 |
| **Previous diagnosis with other cancer (%)** | 13 (10.3) | 7 (9.3) | 6 (11.8) | 0.660 |
| **Family history of gastric cancer (%)** | 37 (29.4) | 23 (30.7) | 14 (27.5) | 0.697 |
| ***H. pylori* infection (%)** | 99 (78.6) | 57 (76.0) | 42 (82.4) | 0.394 |
| **Current or ex-smoker (%)** | 84 (66.7) | 59 (78.7) | 25 (49.0) | 0.001 |
| **Previous abnormality at the same location (%)** | 10 (7.9) | 3 (4.0) | 7 (13.7) | 0.089 |
| **Gastric atrophy, Kimura-Takemoto classification (%)** |  |  |  | 0.001 |
| **Absent (C0)** | 5 (4.0) | 0 (0.0) | 5 (9.8) |  |
| **Mild (C1, C2)** | 25 (19.8) | 9 (12.0) | 16 (31.4) |  |
| **Moderate (C3, O1)** | 75 (59.5) | 51 (68.0) | 24 (47.1) |  |
| **Severe (O2, O3)** | 21 (16.7) | 15 (20.0) | 6 (11.8) |  |
| **Intestinal metaplasia**^‡^ **(%)** | 75 (59.5) | 56 (74.7) | 19 (37.3) | 0.001 |
| **Interval since last upper GI endoscopy, median (IQR) (months)** | 12.0 (11.0-14.3) | 12.0 (11.0-15.0) | 12.0 (11.0-14.0) | 0.874 |
| **Size, median (IQR) (cm)** | 1.30 (0.70-1.85) | 0.80 (0.60-1.40) | 2.00 (1.40-3.00) | <0.001 |
| **Ulcer (%)** | 24 (19.0) | 8 (10.7) | 16 (31.4) | 0.004 |
| **Undifferentiated histology (%)** | 40 (31.7) | 0 (0.0) | 40 (78.4) | - |
| **Submucosal invasion**^§^ **(%)** | 14/111 (12.6) | 1/71 (1.4) | 13/40 (32.5) | <0.001 |
| **Location (%)** |  |  |  | <0.001 |
| **Upper third** | 30 (23.8) | 11 (14.7) | 19 (37.3) |  |
| **Middle third** | 30 (23.8) | 13 (17.3) | 17 (33.3) |  |
| **Lower third** | 66 (52.4) | 51 (68.0) | 15 (29.4) |  |

ET-IGC, endoscopically treatable gastric cancer; EUT-IGC, endoscopically untreatable gastric cancer; SD, standard deviation; BMI, body mass index; IQR, interquartile range; *H. pylori*, *Helicobacter pylori*; GI, gastrointestinal.

^†^ Comparison between ET-IGC and EUT-IGC.

^‡^ Endoscopically diagnosed intestinal metaplasia

^§^ Some denominators do not match the total numbers because of missing data.

Continuous variables are presented as the mean ± standard deviation or median with interquartile range according to fitness of normal distribution and categorical variables are presented as numbers and effective percentage excluding missing data.

**Supplementary table 2. Baseline and pathologic characteristics of interval gastric neoplasms detected since 2010**

|  | **Overall**  **(n = 204)** | **ET-IGN group**  **(n = 170)** | **EUT-IGN group**  **(n = 34)** | ***p***^†^ |
| --- | --- | --- | --- | --- |
| **Age, mean ± SD (yr)** | 59.5 ± 9.2 | 60.5 ± 9.0 | 54.6 ± 8.8 | 0.001 |
| **Male sex (%)** | 152 (74.5) | 131 (77.1) | 21 (61.8) | 0.062 |
| **BMI, median (IQR) (kg/m^2^)** | 24.2 (22.3-26.0) | 24.5 (22.5-26.0) | 23.2 (21.2-24.3) | 0.087 |
| **Previous diagnosis with other cancer (%)** | 28 (13.7) | 24 (14.1) | 4 (11.8) | 1.000 |
| **Family history of gastric cancer (%)** | 47 (23.0) | 35 (20.6) | 12 (35.3) | 0.063 |
| ***H. pylori* infection**^‡^ **(%)** | 149/203 (73.4) | 120/169 (71.0) | 29/34 (85.3) | 0.063 |
| **Current or ex-smoker (%)** | 133 (65.2) | 115 (67.6) | 18 (52.9) | 0.100 |
| **Previous abnormality at the same location (%)** | 23 (11.3) | 20 (11.8) | 3 (8.8) | 0.773 |
| **Gastric atrophy, Kimura-Takemoto classification (%)** |  |  |  | 0.013 |
| **Absent (C0)** | 8 (3.9) | 5 (2.9) | 3 (8.8) |  |
| **Mild (C1, C2)** | 34 (16.7) | 23 (13.5) | 11 (32.4) |  |
| **Moderate (C3, O1)** | 124 (60.8) | 108 (63.5) | 16 (47.1) |  |
| **Severe (O2, O3)** | 38 (18.6) | 34 (20.0) | 4 (11.8) |  |
| **Intestinal metaplasia**^§^ **(%)** | 119 (58.3) | 107 (62.9) | 12 (35.3) | 0.003 |
| **Interval since last upper GI endoscopy, median (IQR) (months)** | 12.0 (11.0-14.0) | 12.0 (11.0-14.0) | 12.0 (11.8-13.3) | 0.753 |
| **Size, median (IQR) (cm)** | 0.80 (0.60-1.60) | 0.80 (0.50-1.20) | 2.00 (1.23-2.80) | <0.001 |
| **Ulcer (%)** | 15 (7.4) | 5 (2.9) | 10 (29.4) | <0.001 |
| **Undifferentiated histology (%)** | 28 (13.7) | 0 (0.0) | 28 (82.4) | - |
| **Submucosal invasion**^‡^ **(%)** | 8/197 (4.1) | 1/169 (0.6) | 7/28 (25.0) | <0.001 |
| **Location (%)** |  |  |  | 0.005 |
| **Upper third** | 39 (19.1) | 26 (15.3) | 13 (38.2) |  |
| **Middle third** | 53 (26.0) | 44 (25.9) | 9 (26.5) |  |
| **Lower third** | 112 (54.9) | 100 (58.8) | 12 (35.3) |  |

ET-IGCN, endoscopically treatable gastric cancer; EUT-IGN, endoscopically untreatable gastric cancer; SD, standard deviation; BMI, body mass index; IQR, interquartile range; *H. pylori*, *Helicobacter pylori*; GI, gastrointestinal.

^†^ Comparison between ET-IGN and EUT-IGN.

^‡^ Some denominators do not match the total numbers because of missing data.

^§^ Endoscopically diagnosed intestinal metaplasia

Continuous variables are presented as the mean ± standard deviation or median with interquartile range according to fitness of normal distribution and categorical variables are presented as numbers and effective percentage excluding missing data.
