# Supplementary figures and images for: Characteristics of interval gastric neoplasms detected within two years after negative screening endoscopy among Koreans
Source: BMC Cancer. 2021 Mar 2;21:218. doi: 10.1186/s12885-021-07929-y (PMC7923316; doi:10.1186/s12885-021-07929-y)

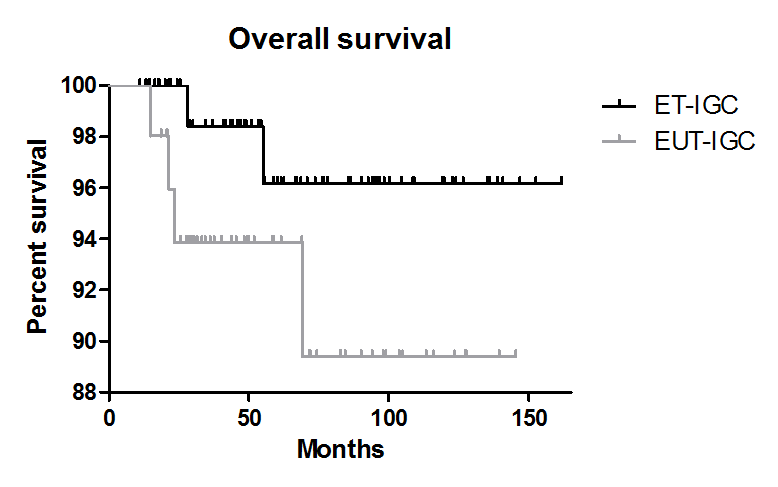

Supplement: Supplementary file 2 — Additional file 2 Table S1. Baseline and pathologic characteristics of interval gastric cancer. Table S2. Baseline and pathologic characteristics of interval gastric neoplasms detected since 2010. [file 12885_2021_7929_MOESM2_ESM.zip › Suppl Fig 1AR3_20210114.tif]

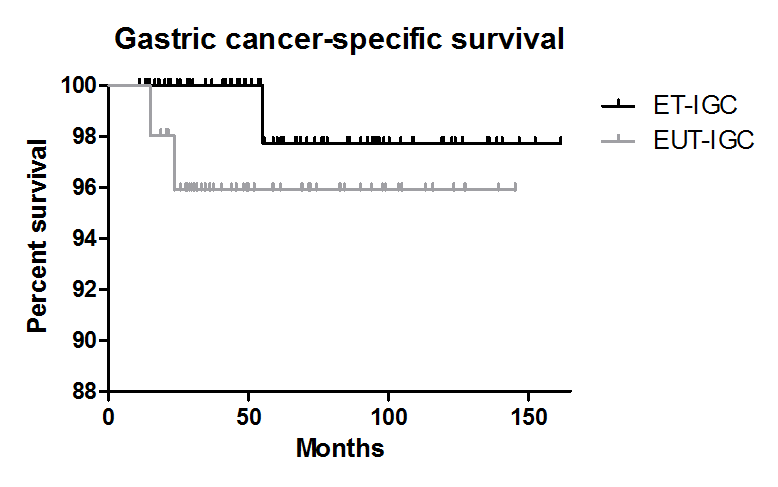

Supplement: Supplementary file 2 — Additional file 2 Table S1. Baseline and pathologic characteristics of interval gastric cancer. Table S2. Baseline and pathologic characteristics of interval gastric neoplasms detected since 2010. [file 12885_2021_7929_MOESM2_ESM.zip › Suppl Fig 1BR3_20210114.tif]
